# *Staphylococcus aureus*: A Review of the Pathogenesis and Virulence Mechanisms

**DOI:** 10.3390/antibiotics14050470

**Published:** 2025-05-06

**Authors:** Rahima Touaitia, Assia Mairi, Nasir Adam Ibrahim, Nosiba S. Basher, Takfarinas Idres, Abdelaziz Touati

**Affiliations:** 1 Department of Natural and Life Sciences, Faculty of Exact Sciences and Natural and Life Sciences, University of Tebessa, Tebessa 12002, Algeria; rahima.touaitia@univ-tebessa.dz; 2 Laboratoire d’Ecologie Microbienne, Faculté des Sciences de la Nature et de la Vie (FSNV), Université de Bejaia, Bejaia 06000, Algeria; assia.mairi@univ-bejaia.dz (A.M.); abdelaziz.touati@univ-bejaia.dz (A.T.); 3 Department of Biology, College of Science, Imam Mohammad Ibn Saud Islamic University (IMSIU), Riyadh 13318, Saudi Arabia; nsbasher@imamu.edu.sa; 4 Research Laboratory for Management of Local Animal Resources, Rabie Bouchama National Veterinary School of Algiers, Issad ABBAS Street, BP 161 Oued Semar, Algiers 16059, Algeria; t.idres@ensv.dz

**Keywords:** *Staphylococcus aureus*, virulence factors, antibiotic resistance, MRSA, biofilm formation, colonization dynamics, metabolic adaptation

## Abstract

*Staphylococcus aureus* is a formidable human pathogen responsible for infections ranging from superficial skin lesions to life-threatening systemic diseases. This review synthesizes current knowledge on its pathogenesis, emphasizing colonization dynamics, virulence mechanisms, biofilm formation, and antibiotic resistance. By analyzing studies from PubMed, Scopus, and Web of Science, we highlight the pathogen’s adaptability, driven by surface adhesins (e.g., ClfB, SasG), secreted toxins (e.g., PVL, TSST-1), and metabolic flexibility in iron acquisition and amino acid utilization. Nasal, skin, and oropharyngeal colonization are reservoirs for invasive infections, with biofilm persistence and horizontal gene transfer exacerbating antimicrobial resistance, particularly in methicillin-resistant *S. aureus* (MRSA). The review underscores the clinical challenges of multidrug-resistant strains, including vancomycin resistance and decolonization strategies’ failure to target single anatomical sites. Key discussions address host–microbiome interactions, immune evasion tactics, and the limitations of current therapies. Future directions advocate for novel anti-virulence therapies, multi-epitope vaccines, and AI-driven diagnostics to combat evolving resistance. Strengthening global surveillance and interdisciplinary collaboration is critical to mitigating the public health burden of *S. aureus*.

## 1. Introduction

*Staphylococcus aureus* is a versatile Gram-positive coccus, a facultative aero-anaerobic bacterium that is both a commensal organism and an opportunistic pathogen, capable of causing a broad spectrum of human diseases [1]. As one of the most clinically significant bacterial pathogens, it is responsible for infections ranging from superficial skin and (SSTIs) to life-threatening conditions such as pneumonia, endocarditis, osteomyelitis, septic arthritis, and bacteremia [2]. Despite colonizing the anterior nares, skin, and mucosal surfaces in approximately 30% of healthy individuals, *S. aureus* exploits breaches in host barriers or immune defenses to establish invasive infections, underscoring its adaptability and resilience [3].

The pathogenic success of *S. aureus* is primarily attributed to its extensive virulence factors, enabling it to adhere to host tissues, evade immune responses, and damage host cells [4]. Surface proteins such as clumping factors (ClfA, ClfB) and fibronectin-binding proteins facilitate adhesion to host cells and tissues. At the same time, secreted toxins like α-hemolysin, Panton-Valentine leukocidin (PVL), and toxic shock syndrome toxin-1 (TSST-1) contribute to tissue destruction and systemic toxicity [5]. Furthermore, its ability to form robust biofilms on medical devices and prosthetic implants enhances persistence by shielding bacteria from antimicrobial agents and host defenses, driving chronic and recurrent infections [6].

The clinical management of *S. aureus* infections is further complicated by its extraordinary capacity to acquire and develop antibiotic resistance. The emergence of methicillin-resistant *S. aureus* (MRSA) in both healthcare and community settings has rendered many first-line antibiotics ineffective, leading to higher morbidity and mortality rates globally [7]. In recent decades, strains with reduced susceptibility to vancomycin (VRSA) and resistance to last-resort antibiotics highlighted the pathogen’s evolutionary adaptability [8]. Understanding the genetic and molecular mechanisms driving antibiotic resistance and the evolutionary dynamics of resistant strains is essential for developing novel therapeutic approaches and public health strategies [9,10].

Given its clinical significance and the urgent challenges posed by antimicrobial resistance (AMR), *S. aureus* remains a critical global research focus. This review comprehensively explores the biology of *S. aureus*, integrating its pathogenesis, virulence factors, and biofilm formation. Additionally, we examine the epidemiological landscape of infections and highlight promising avenues for future research.

## 2. Pathogenesis of *S. aureus*

*S. aureus* is a highly adaptable and opportunistic pathogen that can colonize various anatomical sites, evade the host immune system, and cause a broad spectrum of infections. Its pathogenic potential is driven by a complex interplay of virulence factors that facilitate adherence, invasion, immune evasion, and tissue destruction [11].

### 2.1. Colonization Dynamics

**Figure 1:**
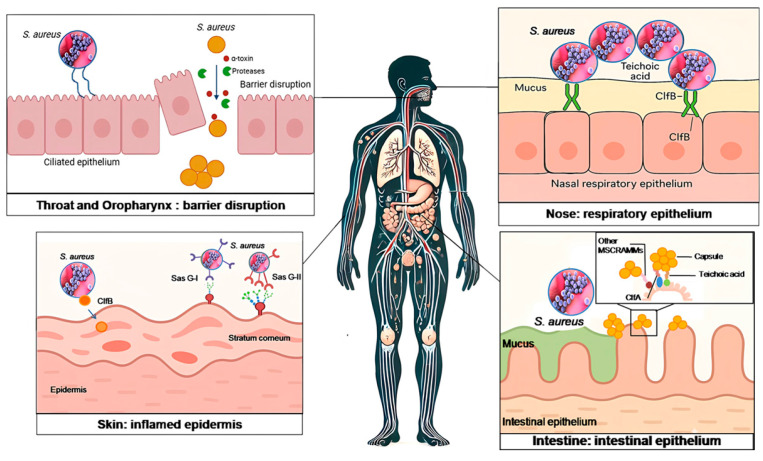
Host–Epithelium Interaction with *S. aureus*: mechanisms of colonization and barrier disruption. **Legend: Throat and Oropharynx:** *S. aureus* adheres to the ciliated epithelium, leading to barrier disruption and potential infection. **Nasal Epithelium:** *S. aureus* colonizes the nasal mucosa, aided by teichoic acid and microbial surface components recognizing adhesive matrix molecules (CHiB). **Skin:** The bacterium interacts with the epidermis, using adhesins like SasG and ClfB to bind to skin components and cause inflammation. **Intestinal Epithelium:** *S. aureus* adheres to the intestinal mucosa, producing toxins and mucus-associated proteins that facilitate colonization.

Colonization of the human body is the first and most crucial step in *S. aureus* pathogenesis, serving as a reservoir for potential infections (Figure 1). Epidemiological studies reveal a nuanced colonization landscape, where approximately 30% of individuals are persistently colonized, serving as long-term reservoirs for recurrent infections, particularly in immunocompromised hosts. An additional 30% undergo intermittent colonization, driven by factors such as bacterial strain adaptability, environmental exposures, and fluctuations in host immunity [11]. The remaining 40% are classified as non-carriers, though transient colonization remains possible under conducive conditions, such as disrupted skin barriers or antibiotic-mediated microbiome shifts [12]. The clinical significance of colonization lies in its role as a precursor to invasive disease. Persistent carriers, for instance, face elevated risks of bacteremia or surgical site infections as colonizing strains exploit breaches in immune or anatomical defenses. Common colonization sites, including the anterior nares, skin, and pharynx, serve as strategic footholds, highlighting the bacterium’s tropism for niches where host–pathogen interactions dictate infection trajectories [13,14].

This dynamic underscores the importance of understanding microbial mechanisms and host-specific vulnerabilities to mitigate colonization-to-infection progression in at-risk populations.

#### 2.1.1. Nasal Cavity

Approximately 20% of the human population are persistent carriers of *S. aureus*, 60% exhibit intermittent carriage, and the remaining 20% show no detectable colonization. The anterior nares constitute the primary ecological niche for this bacterium, with the nasal vestibule’s squamous epithelium serving as the predominant colonization site [3]. Specifically, *S. aureus* adheres to cytokeratin 10, a structural protein expressed in nasal epithelial cells, a key mechanism enabling persistent colonization [15].

Nasal colonization by *S. aureus* is a complex process mediated by microbial interactions, primarily driven by siderophore production, facilitating iron acquisition. *S. aureus* synthesizes two siderophores, staphyloferrin A (SF-A) and staphyloferrin B (SF-B), which can support the growth of other nasal bacteria [16]. Under iron-restricted conditions, wild-type *S. aureus* and single mutants (deficient in either SF-A or SF-B) enhanced the proliferation of commensal bacteria, whereas a double mutant strain (lacking both siderophores) could not support such growth. While most Staphylococcal species, except *Staphylococcus hominis*, exhibited growth benefits from SF-A, *Staphylococcus lugdunensis* uniquely exploited SF-A and SF-B [17,18]. Notably, nearly all *Corynebacterium* isolates demonstrated enhanced growth in response to staphyloferrin activity, underscoring the pivotal role of *S. aureus* in modulating nasal microbial communities [19]. Other bacteria, including *Bacillus cereus*, *Citrobacter koseri*, and *Cutibacterium acnes*, also enhanced growth. These observations indicate that staphyloferrins function as critically essential sources within the nasal niche, directly shaping microbial equilibrium and colonization patterns [20].

##### Host–Microbiome Interactions

Host immune responses also play a crucial role in controlling *S. aureus* nasal colonization. The nasal epithelium produces antimicrobial peptides such as human β-defensins (hBDs) and cathelicidins, which help limit bacterial colonization. *S. aureus* must evade these defenses to persist in the nasal niche. Some strains produce staphylokinase, an enzyme that degrades antimicrobial peptides, reducing host immune clearance [21,22]. Moreover, *S. aureus* modifies its surface charge through the MprF protein, enabling it to repel cationic antimicrobial peptides like LL-37, thereby enhancing its survival in the nasal cavity [23]. Interindividual variation in host immune responses, particularly in β-defensin production, has been associated with differences in *S. aureus* colonization patterns [24].

##### Risk Factors for Nasal Colonization

Nasal colonization by *S. aureus* is a well-documented risk factor for various infections, particularly among surgical patients, immunocompromised individuals, and those with chronic conditions. Surgical site infections are significantly more common in nasal carriers, especially among orthopedic surgery patients with prosthetic implants [15]. Similarly, hemodialysis and peritoneal dialysis patients exhibit a higher nasal carriage rate, correlating with an increased risk of bloodstream infections [25]. In HIV-positive individuals, colonization rates are elevated, leading to a greater likelihood of invasive infections due to their compromised immune status [26,27]. Within the general population, *S. aureus* nasal carriage is linked to skin and soft tissue infections, with studies reporting higher prevalence among individuals with recent antibiotic use [28]. Social interactions also play a key role in transmission dynamics, as close contact with carriers increases colonization risk [29]. Given the risks, targeted screening and decolonization protocols are critical for mitigating infections and curbing transmission in high-risk groups [30].

Nasal colonization rates of MRSA and methicillin-susceptible *S. aureus* (MSSA) vary across different populations and settings. In the general community, approximately 12% to 30% of individuals are colonized with MSSA, whereas MRSA colonization is less common, affecting about 1% to 3% of the population [31]. Specific groups, such as healthcare workers and residents of long-term care facilities, often exhibit higher MRSA colonization rates due to increased exposure. For instance, studies have reported MRSA carriage rates of up to 7% in hospital settings and up to 2% in the community [32].

Additionally, vulnerable populations, including people experiencing homelessness, may experience elevated colonization rates; a study in Lisbon, Portugal, found a 51.2% MSSA carriage rate and a 1.2% MRSA carriage rate among homeless individuals [33].

Nasal carriage rates of MRSA and MSSA exhibit significant variability across countries and populations, reflecting the interplay of environmental factors, healthcare exposure, socioeconomic conditions, and regional infection control practices (Table 1). In the United Kingdom, orthopedic outpatients demonstrated MSSA colonization rates ranging from 22.4% to 35.6%, while MRSA prevalence remained relatively low (1.2–4.3%) [34]. Similarly, patients undergoing fracture fixation in the United States showed a 20.18% MSSA and 4.70% MRSA carriage rate [35]. Notably, critically ill pediatric patients in the U.S. had a 6.5% MRSA prevalence, though MSSA rates were not specified [36].

Ethiopian studies highlighted stark contrasts between populations: hospital janitors exhibited higher colonization rates (22.2% MSSA, 8.1% MRSA) compared to non-hospital janitors (14.4% MSSA, 1.4% MRSA) [32]. In Argentina, healthcare workers had a 23.7% MSSA and 6.3% MRSA prevalence [37], whereas France reported markedly lower MRSA rates among healthy blood donors (0.3% MRSA, 29.3% MSSA) and hospitalized patients (1.1% MRSA, 20.2% MSSA) [38].

Community-based studies revealed extremes: Sierra Leone’s general population showed alarmingly high rates (42.7% MSSA, 14% MRSA) [39], while Lebanon reported 38.4% MSSA and a low 1.6% MRSA [40]. In China, medical students displayed moderate colonization (15.4–23.1% MSSA, 3.0–9.4% MRSA) [41].

High-risk groups stood out: Saudi Arabian healthcare workers had 40% MSSA and 18% MRSA rates [42], and Algerian livestock workers showed 50% MSSA in humans and 7.6% in livestock [43]. Conversely, homeless individuals in Lisbon, Portugal, had a striking 50% MSSA rate but minimal MRSA (1.2%) [33].

These disparities underscore the profound impact of healthcare access, occupational exposure, socioeconomic inequality, and regional public health strategies on *S. aureus* colonization dynamics. Populations with frequent healthcare interactions or limited resources often face elevated MRSA risks, emphasizing the need for targeted infection control measures and surveillance.

#### 2.1.2. Skin Colonization

*S. aureus* preferentially colonizes moist areas of the skin rich in sebaceous glands, such as the axilla, groin, and perineum [14]. This colonization is facilitated by the bacterium’s ability to adhere to corneocytes, the outermost skin cells, through specific surface proteins like Clumping factor B (ClfB) and surface protein G (SasG) [44]. These adhesins facilitate binding to host proteins like loricrin, involucrin, and cytokeratin, which are abundant in the skin’s cornified layer [45].

The skin’s unique microbiome and physicochemical properties create niche-specific challenges. ClfB and SasG act as molecular bridges, exploiting skin desquamation processes to establish persistent colonization even under hygienic conditions [46].

##### Role of Clumping Factor B (ClfB)

ClfB, a cell wall-anchored protein, binds to loricrin, a significant component of the cornified envelope in corneocytes. This interaction enhances *S. aureus* adherence to the skin, promoting colonization [44]. Studies have shown that ClfB-deficient strains exhibit reduced binding to human corneocytes, highlighting its importance in colonization [47]. ClfB-deficient mutants show reduced bacterial burden and milder pathology in skin abscess models, indicating its importance in disease progression. Vaccines targeting ClfB have demonstrated protective effects in preclinical studies, reducing bacterial burden and skin pathology [48].

Beyond mechanical adhesion, ClfB modulates host inflammatory responses by interfering with complement activation. This dual function—adhesion and immune evasion—explains why ClfB-knockout strains trigger stronger neutrophil recruitment [49].

##### Role of Surface Protein G (SasG)

SasG is another critical adhesin that mediates *S. aureus’s* attachment to corneocytes. Recent research has identified two major SasG alleles, SasG-I and SasG-II. Structural analyses reveal that SasG-II possesses a unique non-aromatic arginine in its lectin subdomain, enabling it to bind a broader range of ligands than SasG-I. This adaptation allows *S. aureus* strains expressing SasG-II to adhere more effectively to diverse skin environments, conferring a colonization advantage [50].

SasG-II’s structural flexibility reflects evolutionary pressure for host adaptation. Its arginine-rich subdomain enhances binding versatility, providing a competitive edge in heterogeneous skin environments [51].

##### Immune Evasion and Vaccine Challenges

The development of an effective *S. aureus* vaccine has been challenging due to a limited understanding of the immune mechanisms that confer protection against the pathogen [52]. While Th17 cells and interleukin-17 (IL-17) are critical for protective immunity, *S. aureus* counteracts these defenses by modulating Toll-like receptor (TLR) signaling and producing superantigens [53,54]. Additionally, antigenic redundancy (e.g., compensatory adhesins replacing ClfB/SasG) and the pathogen’s ability to skew immune responses toward non-protective antibody subtypes contribute to vaccine failures [55].

Recent strategies focus on multi-epitope vaccines targeting both adhesins (e.g., ClfB, SasG) and toxins (e.g., alpha-hemolysin) combined with adjuvants that enhance mucosal and cutaneous immunity [54].

#### 2.1.3. Throat and Oropharynx Colonization

While nasal colonization is well-studied, emerging evidence highlights the oropharynx (throat) as a critical yet underrecognized reservoir for *S. aureus*, including MRSA. Understanding its prevalence, persistence, and role in transmission is vital for infection control and clinical management.

##### Prevalence of S. aureus in the Throat/Oropharynx

The prevalence of *S. aureus* in the throat and oropharynx has gained attention as an essential consideration in studying bacterial colonization in humans. Recent research indicates that the throat may exhibit higher carriage rates of *S. aureus* than the nasal passages. Studies conducted by various authors reveal that a significant proportion of patients and healthcare workers carry *S. aureus* primarily in their throats [12].

A notable study by Nilsson and Ripa) demonstrated that the rate of throat carriage among patients significantly exceeded that of nasal carriage, showing figures of 40% versus 31% [56]. Additionally, their research pointed out that a notable percentage of individuals were identified as exclusive throat carriers. Mertz et al. found that 30.2% of community participants had exclusive throat carriage of *S. aureus*, compared to 18.4% among hospitalized patients and healthcare workers [57]. Further investigation in a cohort of healthy individuals by Hamdan-Partida et al. confirmed this trend, showing that *S. aureus* was more frequently isolated from throat samples (46.5%) than from nasal samples (37.1%), and among the colonized individuals, as many as 38% of the people were exclusive throat carriers [58].

Hanson et al. found a correlation between colonization at both sites, with oropharyngeal swabs showing higher sensitivity for detection (77.27%) than nasal swabs (72.75%) in their 2017 study [59]. A subsequent study reported that the sensitivity of oropharyngeal cultures (86.1%) was notably higher than that of nasal cultures (58.2%) [60].

The prevalence of MRSA and MSSA in nasal and throat samples varies significantly across different populations and studies. Research has shown that throat samples yield higher rates of MRSA isolation than nasal samples in some populations. While in other studies, especially in hospital settings, the reverse may be true, throat samples show more significant MRSA colonization. According to a report by Hamdan-Partida et al., MRSA was detected more often in positive nasal samples (32.9%) than in positive throat samples (23%) within the general population [58]. Conversely, a study published by Senn et al. revealed that MRSA was more often detected in throat swabs (with a positivity rate of 15%) compared to nasal swabs (12% positive for MRSA) [61]. Similarly, Marshall and Spelman previously found that 69.2% of patients tested positive in nasal samples, compared to 71.4% in throat samples. The research assessed whether the throat could detect several additional MRSA-colonized patients not identified through sampling at keratinized skin sites (such as the anterior nares, perineum, and axillae). The study found that throat samples demonstrated greater sensitivity than pooled keratinized skin samples, with rates of 76% versus 60% [62].

##### Persistence and Challenges in Eradication

The persistence of *S. aureus* in the oropharynx poses significant clinical challenges, often outstripping the transient nature of nasal colonization. *S. aureus* throat colonization persists longer than nasal carriage due to biofilm formation, AMR, and asymptomatic shedding [63].

The throat’s tonsillar crypts enable *S. aureus* to form biofilms, allowing adherence to surfaces and resistance to clearance mechanisms. Components like polysaccharide intercellular adhesin (PIA) and surface proteins aid in adhesion and immune evasion [64]. Biofilms also protect *S. aureus* from antibiotics, creating “persister” cells that survive treatment. Biofilm-forming strains are 3–5 times more likely to persist in the throat, complicating eradication efforts [6].

Oropharyngeal *S. aureus* isolates often show higher AMR rates than nasal strains, primarily due to horizontal gene transfer from co-colonizing bacteria (e.g., MRSA with *mecA* or macrolide resistance). MRSA strains in the throat frequently harbor additional resistance genes, complicating treatment options [65]. Furthermore, *S. aureus* exhibits significant phenotypic heterogeneity, with some subpopulations expressing efflux pumps to expel antiseptics, while others mutate penicillin-binding proteins to evade beta-lactams. This genetic diversity complicates targeted therapy and increases the risk of treatment failures, necessitating vigilance from clinicians in managing infections caused by *S. aureus* in oropharyngeal settings [32].

Unlike symptomatic infections (e.g., pharyngitis), oropharyngeal colonization is typically asymptomatic, allowing carriers to unknowingly sustain and spread *S. aureus* [66]. Longitudinal studies reveal that throat colonization can persist for over a year in 10–20% of individuals, even in the absence of nasal carriage [12]. This silent reservoir facilitates community and hospital transmission as carriers shed bacteria through respiratory droplets, saliva, or contaminated hands. Alarmingly, standard decolonization protocols such as nasal mupirocin ointment or topical antiseptics often fail to eliminate throat colonization. For instance, a meta-analysis found that nasal mupirocin reduced nasal MRSA by 50% but did not significantly impact throat carriage, highlighting the need for site-specific interventions [31].

#### 2.1.4. Gastrointestinal/Perineum and Urogenital Tract Colonization

*S. aureus* is primarily known for colonizing the nasal passages, but it can also inhabit the gastrointestinal (GI) tract, especially in hospitalized patients and those undergoing antibiotic treatment [66]. This colonization has significant clinical implications [67]. Studies have shown that hospitalized individuals are at a higher risk of GI colonization by *S. aureus*. For instance, research indicates intestinal colonization among these patients increases the risk of subsequent infections and may contribute to nosocomial transmission [68,69,70].

Antibiotics can disrupt the normal gut microbiota, creating an environment conducive to *S. aureus* colonization. This disruption reduces microbial competition, allowing *S. aureus*, including MRSA, to establish itself in the GI tract [71]. GI colonization by *S. aureus* has been associated with an increased risk of developing infections, particularly in immunocompromised patients. The presence of *S. aureus* in the intestines can serve as a reservoir for invasive infections [72]. Patients with GI colonization can shed *S. aureus* into their surroundings, contaminating surfaces and medical equipment. This environmental contamination risks transmission to other patients and healthcare workers, potentially leading to outbreaks within healthcare settings [69].

*S. aureus* frequently colonizes the perineal region in both males and females, with a notable presence in the vaginal and rectal mucosa. This colonization poses significant risks for postpartum infections [73]. Deng et al. studied *S. aureus* colonization in the vaginal tract, which can cause postpartum infections [74]. Their murine model revealed that *S. aureus* utilizes fibrinogen-binding adhesins for adherence and iron acquisition, thereby facilitating survival. The pathogen also upregulates immune evasion genes to resist neutrophil clearance. Mutants lacking adhesion or iron uptake showed significantly reduced colonization, highlighting the crucial roles of these factors [74].

These findings suggest that targeting bacterial adhesins or iron metabolism could offer new strategies to prevent *S. aureus* vaginal colonization and reduce maternal and neonatal infection risks [74].

A recent study investigated *S. aureus* colonization in the female lower genital tract among women in labor, assessing the presence of virulence genes and methicillin resistance. Researchers analyzed 85 *S. aureus* isolates from vaginal swabs, finding that 15.3% carried at least one virulence gene, with 10.6% harboring the *pvl* gene. Additionally, 55.3% of isolates were MRSA, though most strains lacked virulence genes. The findings indicate a potential risk of neonatal transmission during childbirth, emphasizing the need for improved infection control measures in maternal healthcare settings [73].

##### Detection and Decolonization

Routine screening for *S. aureus* colonization typically focuses on nasal swabs. However, considering the prevalence of GI colonization, incorporating rectal swabs into screening protocols can improve detection rates, ensuring that colonized individuals are identified and managed appropriately [69,75]. Implementing decolonization protocols, such as chlorhexidine baths and intranasal mupirocin, has effectively reduced the rates of *S. aureus* colonization and subsequent infections. Targeted and universal decolonization approaches have effectively decreased transmission within healthcare facilities [76].

### 2.2. Virulence Factors of S. aureus

*S. aureus* remains a formidable human pathogen, capable of causing a spectrum of infections, ranging from superficial skin abscesses to life-threatening conditions such as bacteremia, endocarditis, and toxic shock syndrome [77]. Its remarkable adaptability and pathogenic success are primarily attributed to a sophisticated arsenal of virulence factors [78].

These factors, encompassing surface proteins, secreted toxins, and metabolic adaptations, enable *S. aureus* to colonize host tissues, evade immune defenses, and inflict cellular damage [79]. Research has illuminated the intricate interplay between these virulence determinants, revealing a dynamic and context-dependent expression profile contributing to the pathogen’s versatility [80]. Understanding the molecular mechanisms underlying *S. aureus* virulence is crucial for developing effective therapeutic strategies and combating the growing challenge of antibiotic resistance [77].

#### 2.2.1. Surface Proteins and Adhesion

*S. aureus* is a versatile pathogen responsible for surface skin lesions to potentially mortal conditions, such as sepsis and pneumonia [81]. A critical aspect of *S. aureus*’s pathogenicity is its ability to adhere to host tissues, a process facilitated mainly by a group of proteins expressed on the surface known as microbial surface components that recognize the molecules of the adhesive matrix (MSCRAMM) [77]. The MSCRAMMs are specialized adhesins that bind to several elements of the extracellular matrix (ECM), including fibrinogen, fibronectin, and collagen, thus playing a central role in infection’s initial stages and biofilm formation [82].

The structure and function of MSCRAMM have been studied widely, revealing their importance in the bacterial life cycle and host–pathogen interactions. Each MSCRAMM is characterized by its ability to join specific ECM components, facilitating bacterial adhesion and allowing *S. aureus* to avoid the host’s immune responses [45]. For example, fibronectin-binding proteins (FNBP) are among the very well-characterized MSCRMS, promoting the union of *S. aureus* to host and biomaterial cells through fibronectin, a key component in the healing and regeneration of tissues [83]. MSCRAMM, such as Factor A (Clfa) and protein A, also play fundamental roles in adherence and immune evasion by binding to fibrinogen and host immunoglobulins [84]. These interactions highlight the double functionality of the MSCRAMM to promote bacterial colonization and simultaneously hinder immune defense mechanisms [11].

Regarding pathogenesis, the attachment of S. aureus to the tissues of the host through MSCRAMMs facilitates the formation of biofilms, a critical factor in chronic infections [6,85]. In addition, infections associated with biofilm are notoriously difficult to eradicate, underlining the need for a deeper understanding of MSCRAMMs in the biology of *S. aureus* [86]. The incorporation of MSCRAMMs into the biofilm structure not only enhances bacterial persistence but also increases the pathogenicity of S. aureus strains, particularly in infections related to implants and bloodstream infections associated with catheters [78,87].

Given the central role of MSCRAMMs in adherence and pathogenesis, they have become possible therapeutic objectives for infection prevention. Strategies targeting these adhesins involve the development of vaccines that induce protective immunity against MSCRAMMs or the design of small molecules that inhibit their function. For example, peptide-based inhibitors have proven promising in interrupting the FNBP union with fibronectin, thereby reducing bacterial adhesion and in vitro biofilms [88]. Additionally, monoclonal antibodies (mABs) targeting specific MSCRAMMs could enhance opsonophagocytosis and facilitate the immune system’s elimination of S. aureus [89].

#### 2.2.2. Secreted Toxins and Immune Evasion

Beyond surface adhesion, *S. aureus* employs a potent arsenal of secreted toxins to disrupt host cellular functions and evade immune clearance [90]. These toxins, including cytolysins (e.g., alpha-toxin, PVL, superantigens (e.g., TSST-1), and exfoliative toxins), contribute to tissue damage by cleaving desmosomal cadherins, leading to severe skin conditions such as staphylococcal scalded skin syndrome (SSSS), immune dysregulation, and systemic toxicity [91,92]. *S. aureus* relies on a combination of surface proteins and secreted toxins to establish infections. Biofilm formation enhances bacterial persistence, while superantigens trigger excessive immune activation, leading to cytokine storms that exacerbate disease severity [92]. The pathogen also produces membrane-damaging factors such as hemolysins and cytolytic peptides, which disrupt host cell membranes and facilitate immune evasion [93]. Recent reports have expanded our understanding of the molecular mechanisms by which these toxins interact with host cells, revealing intricate signaling pathways and cellular targets [11,94,95]. For example, research on PVL has demonstrated its ability to form pores in neutrophil membranes, leading to cell lysis and the release of inflammatory mediators [96]. Furthermore, advancements in genomics and transcriptomics have facilitated the identification of novel toxin variants and their association with specific clinical manifestations, providing insights into the evolving virulence landscape of *S. aureus* [11].

Genetic variation among *S. aureus* strains significantly influences their pathogenic potential, mainly through differences in toxin production. This variability is primarily driven by mobile genetic elements (MGEs) such as plasmids, bacteriophages, transposons, and *staphylococcal cassette chromosome* (SCC) elements, which facilitate the horizontal transfer of virulence genes [4]. One of the most clinically relevant toxins, PVL, is encoded by the *lukS-PV* and *lukF-PV* genes, which are carried by bacteriophages such as ΦSa2 and are strongly associated with community-acquired MRSA (CA-MRSA) strains like USA300, contributing to severe skin and *SSTIs* and necrotizing pneumonia [97]. Enterotoxins, including *sea*, *seb*, and *sec*, responsible for staphylococcal food poisoning, are commonly found on *S. aureus* pathogenicity islands (SaPIs), facilitating their dissemination among strains [97]. Similarly, TSST-1, encoded by the *tst* gene, is typically located on SaPI1 and can trigger life-threatening toxic shock syndrome due to its superantigenic properties [98]. Exfoliative toxins, *eta* and *etb*, which cause SSSS, exhibit different genetic locations, with *eta* being chromosomally encoded while *etb* is often carried on a plasmid [99]. This genetic diversity results from horizontal gene transfer, selective pressure from host immunity and antibiotic exposure, and genomic recombination events, making it challenging to predict virulence based solely on genetic profiling [100].

In addition to toxin production, *S. aureus* secretes proteases that target host immune system components. These proteases degrade key immune molecules, weakening the host defense and promoting bacterial survival within tissues. Understanding these enzymatic mechanisms is essential for developing strategies to counteract immune evasion [101].

Recent genomic studies have shed light on the complex network of genes that drive the virulence of S. aureus, revealing a multifaceted strategy that encompasses adhesion, toxin secretion, and immune evasion. These investigations have identified key virulence factors, such as MSCRAMMs, which mediate bacterial adherence to host tissues and serve as the critical first step in establishing infection [49]. Moreover, the ability of *S. aureus* to form biofilms, structured communities of bacterial cells embedded in a self-produced extracellular matrix, is regulated by a suite of genes that enhance bacterial survival, antibiotic resistance, and persistence within the host [102]. In parallel, the coordinated expression of genes responsible for producing cytolytic toxins, including alpha-hemolysin and phenol-soluble modulins, contributes to tissue destruction and subversion of the host immune response [103]. Collectively, this network of virulence determinants underpins the pathogenic success of *S. aureus* and represents promising targets for developing novel therapeutic interventions to mitigate its impact on human health [77].

### 2.3. Staphylococcal PVL: Clinical Implications, Molecular Mechanisms, and Genetic Landscape

#### 2.3.1. Importance of PVL in *S. aureus* Infections

PVL, a bicomponent pore-forming cytotoxin produced by *S. aureus*, is a critical virulence factor implicated in severe community-acquired infections. PVL targets neutrophils, monocytes, and macrophages by binding to human complement receptors C5aR and C5L2, inducing pore formation and necrotic cell death. This selective cytotoxicity facilitates immune evasion, enabling bacterial persistence and tissue invasion [104]. PVL is strongly associated with skin and soft tissue infections (SSTIs), particularly abscesses and furuncles, and is frequently linked to CA-MRSA strains such as the European ST80 clone and USA300 lineage. These strains exhibit heightened transmissibility and are responsible for recurrent SSTIs, with recurrence rates threefold higher than PVL-negative isolates [105,106].

PVL-positive *S. aureus* (PVL-SA) strains are disproportionately associated with necrotizing pneumonia (NP), particularly in young immunocompetent individuals. Mortality rates in NP cases range from 56% to 75%, often following influenza-like prodromes. PVL-SA pneumonia is characterized by rapid progression, hemoptysis, and multilobar infiltrates, necessitating intensive care [107,108]. However, meta-analyses indicate that PVL is less prevalent in invasive infections (e.g., bacteremia, musculoskeletal infections) than SSTIs, challenging its role as a sole marker of invasive disease severity [109].

Epidemiologically, PVL-SA prevalence varies geographically, with rates as high as 74% in Africa compared to ≤10% in Europe. Travel to tropical regions and close-contact settings (e.g., households, athletic facilities) facilitates transmission. High-risk groups include children, intravenous drug users, military personnel, and incarcerated individuals [108,110]. Pediatric populations are particularly vulnerable, with PVL-SA infections frequently involving necrotizing pneumonia, osteomyelitis, and bacteremia. Pulmonary involvement in children predicts intensive care needs (OR 25.35), underscoring the toxin’s aggressive clinical trajectory [111,112].

Despite its clinical significance, PVL’s role remains controversial. Murine models fail to fully recapitulate human-specific receptor interactions, leading to inconsistent correlations between PVL presence and disease severity. Other virulence factors, such as α-hemolysin and phenol-soluble modulins, likely synergize with PVL to drive necrotizing phenotypes [113,114]. Furthermore, PVL-positive MSSA strains are increasingly reported, highlighting that PVL’s pathogenicity extends beyond MRSA [115].

Clinically, PVL-SA infections are underdiagnosed due to non-routine testing. Elevated C-reactive protein with paradoxically low leukocytosis may signal PVL-mediated leukotoxicity. Early microbiological testing, decolonization protocols, and toxin-suppressive therapies (e.g., clindamycin) are critical to mitigating recurrence and systemic complications [108,116].

#### 2.3.2. Mechanism of Action of PVL

PVL is a bicomponent pore-forming cytotoxin produced primarily by MRSA strains, including the highly virulent MRSA-ST80 lineage. Composed of two synergistically acting subunits, LukS-PV and LukF-PV, PVL exerts its cytotoxic effects by targeting human leukocytes, particularly neutrophils, monocytes, and macrophages. The toxin’s mechanism involves sequential binding to host cell receptors, pore formation, and subsequent immune modulation, contributing to severe inflammatory responses and tissue necrosis [117].

LukS-PV initiates cytotoxicity by binding to complement receptors C5aR1 (CD88) and C5L2 on leukocyte membranes, a process mediated by interactions with the N-terminal and core regions of these receptors. LukF-PV subsequently associates with LukS-PV, forming hetero-oligomeric complexes. Structural studies describe these complexes as octameric (four LukS-PV and four LukF-PV subunits) or heptameric pores, with discrepancies attributed to experimental methodologies. The assembled β-barrel pores disrupt membrane integrity, causing rapid ion efflux (e.g., K⁺, Ca^2^⁺), loss of mitochondrial membrane potential, and osmotic lysis. At sublytic concentrations (~5 nM), PVL induces caspase-dependent apoptosis, while higher concentrations (~200 nM) provoke necrotic cell death via overwhelming pore formation [114,117,118].

Beyond direct cytotoxicity, PVL primes neutrophils at sublytic doses, enhancing the oxidative burst and triggering the release of pro-inflammatory mediators such as IL-8, IL-6, leukotriene B4, myeloperoxidase, and lysozyme. This priming, mediated via C5aR1, amplifies local inflammation and tissue damage. Concurrently, LukS-PV is a competitive antagonist of C5a-induced immune activation, impairing calcium mobilization and further dysregulating host defenses. PVL also activates nuclear factor-kappa B (NF-κB) in neutrophils, exacerbating cytokine storms and systemic inflammation, as observed in necrotizing pneumonia and acute respiratory distress syndrome (ARDS) [104,105,113].

PVL synergizes with other virulence determinants, including exfoliative toxin D (etD) and epidermal differentiation inhibitor B (edinB), to enhance tissue invasion and dissemination in MRSA-ST80 [105]. However, its direct pathogenic role remains debated due to inconsistent results in murine models, which lack compatible C5aR isoforms. Murine resistance highlights PVL’s species specificity, driven by structural variations in host receptors. Furthermore, other toxins (e.g., α-hemolysin, phenol-soluble modulins) can replicate PVL’s necrotizing effects, suggesting functional redundancy in *S. aureus* pathogenesis [109,113].

PVL-mediated leukocytolysis disrupts innate immunity, facilitating bacterial survival and progression to necrotizing infections. In necrotizing pneumonia, PVL targets the alveolar epithelium, causing hemorrhage, cavitating lesions, and leukopenia. Systemic complications, including ARDS and multiorgan failure, correlate with toxin-induced hyperinflammation. Chronic infections, such as atopic dermatitis, are perpetuated by PVL-containing extracellular vesicles, which enhance Toll-like receptor activation and cytokine release. These mechanisms underscore PVL’s dual role as a cytolytic toxin and immune modulator [110,119].

#### 2.3.3. Genetics of the PVL in *S. aureus*

The PVL toxin is encoded by two co-transcribed genes, *lukS-PV* and *lukF-PV*, which form the *luk-PV* operon. These genes are located on the temperate bacteriophages of the *Siphoviridae* family, primarily φSa2, integrated into the *S. aureus* genome between the lysis module and the attP attachment site within lysogeny genes [120]. The phage-borne nature of these genes facilitates horizontal transfer via prophage induction or transduction by mobile genetic elements, including staphylococcal pathogenicity islands (SaPIs) [121,122]. At least eight distinct PVL-encoding phages have been identified and classified into three groups based on replication and morphogenesis modules, underscoring their genetic diversity and adaptability [106,123].

Expression of *lukS-PV* and *lukF-PV* is tightly regulated, peaking during the late exponential to stationary growth phases. The *agr* system, particularly agr-I and agr-III alleles, modulates transcription alongside transcription factors MgrA, SarA, and Rot. Environmental triggers, such as β-lactam antibiotics, enhance PVL production by activating the *pvl* promoter and inducing phage replication, thereby amplifying toxin release. Conversely, clindamycin and linezolid suppress toxin synthesis, highlighting the therapeutic implications of antibiotic selection [109,117,124].

PVL genes exhibit a distinct epidemiological distribution. While rare in nasal carriage isolates (0.6–2.1%), they are highly prevalent in strains from skin and SSTIs (38.9%) and necrotizing pneumonia (>90%). Genomic analyses reveal their presence across diverse clonal complexes (CC1, CC5, CC8, CC30, CC45, CC121, ST80) and MRSA or MSSA lineages. Notably, PVL-positive MSSA strains are common in Europe, whereas the MRSA-ST80 clone, frequently carrying SCC*mec* type IVc and agr-III, demonstrates >90% PVL prevalence. PVL-negative ST80 variants retain φSa2 prophage remnants, suggesting ancestral PVL-positive origins [105,107,125].

Horizontal transfer of PVL genes is mediated by phage induction, often triggered by antibiotics such as ciprofloxacin, trimethoprim-sulfamethoxazole, or tobramycin. This mobility underpins the global dissemination of PVL-positive clones, including USA300 (ST8), USA400, and ST59, which frequently co-harbor virulence factors like ACME, *etD*, and *edinB*. Genetic conservation (>95% amino acid identity) and operon stability make *lukSF-PV* attractive targets for vaccines and therapeutics. However, the non-canonical pairing of PVL subunits with other leukocidins (e.g., HlgCB, LukED) in vitro suggests functional versatility, complicating therapeutic strategies [126,127,128].

### 2.4. Metabolic Factors

Beyond traditional virulence factors, *S. aureus* exhibits remarkable metabolic flexibility that significantly contributes to its pathogenicity. The ability to adapt its metabolic pathways to diverse host environments is crucial for its survival and proliferation. Recent research has highlighted the importance of nutrient acquisition, particularly iron and zinc, in the virulence of *S. aureus* [129].

#### 2.4.1. Iron Acquisition by *S. aureus*

Iron is an essential nutrient for *S. aureus* and is critical in bacterial metabolism, replication, and virulence [130]. However, the host employs a defense mechanism known as nutritional immunity, which restricts free iron availability by sequestering it within proteins such as transferrin, lactoferrin, and hemoglobin [131]. *S. aureus* has developed sophisticated iron acquisition strategies to overcome this limitation, primarily by producing siderophores and heme uptake systems [132]. The bacterium synthesizes siderophores, such as staphyloferrin A and staphyloferrin B, high-affinity iron-chelating molecules that are capable of extracting iron from host proteins. These siderophores bind iron tightly and are then recognized by specific membrane receptors, allowing the bacterium to internalize the iron-bound complexes [133]. In addition to siderophore-mediated uptake, *S. aureus* exploits heme as a rich source of iron, utilizing the iron-regulated surface determinant (Isd) system. This system consists of a series of surface receptors (IsdA, IsdB, IsdC) that extract heme from hemoglobin, transport it into the bacterial cytoplasm, and degrade it to release iron [134]. This dual strategy of siderophore production and heme acquisition enables *S. aureus* to thrive in iron-limited environments within the host, enhancing its ability to establish infections and evade immune defenses. Targeting these iron-scavenging mechanisms presents a promising strategy for developing novel antimicrobial therapies [133].

#### 2.4.2. Carbon Metabolism in *S. aureus*

The metabolic flexibility of *S. aureus* is a key factor in its ability to thrive in diverse host environments [135]. Unlike many bacteria that rely on a single primary carbon source, *S. aureus* can metabolize many carbon substrates, including glucose, lactate, and amino acids [51]. This adaptability enables the pathogen to efficiently switch between different energy sources depending on nutrient availability in the host [136]. When glucose is abundant, *S. aureus* primarily uses glycolysis and the pentose phosphate pathway to generate ATP and biosynthetic precursors [137]. However, in glucose-limited conditions, the bacterium shifts to alternative carbon sources such as lactate, which is transported into the cell via the lactate permease and converted into pyruvate for further processing in the tricarboxylic acid (TCA) cycle [137].

Additionally, *S. aureus* can utilize amino acids as carbon and nitrogen sources through deamination and incorporation into central metabolic pathways [136]. This metabolic versatility is particularly advantageous in nutrient-restricted environments, such as infected tissues or biofilm communities, where intense competition for resources occurs. Furthermore, the ability to modulate carbon metabolism influences virulence, as metabolic byproducts can affect host immune responses and bacterial persistence [138].

#### 2.4.3. Amino Acid Metabolism

Recent studies have further elucidated the pivotal role of arginine metabolism in the virulence of *S. aureus*, mainly focusing on the arginine catabolic mobile element (ACME) [51]. ACME is a mobile genetic element that enhances *S. aureus*’s ability to colonize and persist on human skin by conferring resistance to polyamine compounds involved in the host’s immune defense mechanisms [139]. The presence of ACME is notably associated with MRSA strains, particularly the epidemic USA300 clone [140]. This element is uncommon in MSSA strains, suggesting a link between ACME acquisition and increased virulence and antibiotic resistance [141]. The integration of ACME into the *S. aureus* genome is believed to have occurred through horizontal gene transfer from *Staphylococcus epidermidis*, broadening the bacterium’s ability to colonize various niches beyond the nasal cavity, including intact skin. This expansion enhances person-to-person transmission and infection rates [142,143].

Within ACME, the *speG* gene encodes a spermidine acetyltransferase, which plays a pivotal role in mitigating the toxic effects of polyamines on *S. aureus* [144]. By neutralizing these compounds, *S. aureus* can persist on the skin and mucosal surfaces, evade host immune responses, and establish infections more effectively. This mechanism highlights the significance of arginine metabolism and its associated genetic components in the adaptability and virulence of *S. aureus* [144].

Additionally, biofilm formation, a key factor in chronic *S. aureus* infections, is influenced by amino acid metabolism. Genes such as *rocD* and *gudB*, encoding ornithine-oxo-acid transaminase and glutamate dehydrogenase, respectively, are crucial for biofilm development. These enzymes facilitate the utilization of amino acids like glutamine and glutamate, providing metabolic flexibility that supports biofilm maturation and persistence in nutrient-limited environments [145,146].

## 3. Regulation of Virulence Factors

The regulation of virulence in *S. aureus* is a multifaceted process involving a complex network of global regulatory systems that enable the bacterium to adapt to various host environments and evade immune responses. The literature surrounding this topic reveals significant insights into the mechanisms that underpin the pathogenic potential of *S. aureus*.

### 3.1. The Accessory Gene Regulator (agr) System

**Figure 2:**
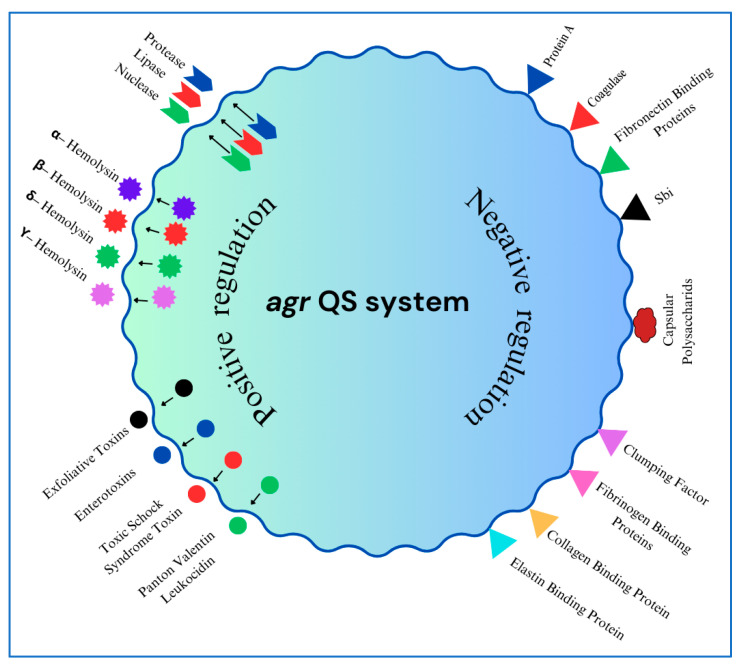
Regulation of virulence factors in *S. aureus* by *agr* QS.

The *agr* quorum-sensing (QS) system in *S. aureus* is pivotal in regulating bacterial behavior during colonization and infection (Figure 2) [147,148,149,150]. Over the span of decades, research has demonstrated its involvement in modulating adhesion, biofilm formation, and virulence, particularly in MRSA strains [151,152]. It comprises a two-component regulatory system activated by an autoinducing peptide (AIP) [153,154,155]. At high bacterial densities, AIP binds to the AgrC histidine kinase receptor, triggering a signaling cascade that leads to the activation of RNAIII. RNAIII regulates the expression of numerous virulence factors, promoting the secretion of toxins (e.g., alpha-toxin, PVL) while repressing surface adhesion proteins, facilitating the transition from a commensal to a pathogenic state [149]. This switch enables S. aureus to transition from an adherent to an invasive lifestyle in response to environmental cues [156]. Comparative analyses of *agr*-like QSs across bacterial species have further elucidated the structural and functional conservation of this regulatory network, providing insights into its evolutionary significance [147].

Recent studies have emphasized the therapeutic potential of targeting the *agr* system to combat biofilm-related infections. Research on QS inhibitors has demonstrated their ability to enhance antibiotic efficacy by disrupting *agr*-mediated signaling [157]. Additionally, the *agr* system plays a crucial role in biofilm formation on medical devices, with RNAIII regulating the expression of virulence factors and surface protein production [158]. Studies on interspecies competition have highlighted the role of AIP signaling in shaping bacterial interactions and pathogenicity [149]. Recent findings have reinforced the significance of the *agr* system in balancing colonization and invasion, making it a promising target for novel antimicrobial strategies [159].

The *agr* QS system in *S. aureus* plays a crucial role in regulating virulence factors by modulating gene expression in response to population density. The system exerts positive and negative regulation on various virulence determinants, influencing the bacterium’s ability to infect and evade host defenses.

**Positive Regulation (+):** The *agr* system upregulates the production of several virulence factors, including Hemolysins (α, β, δ, and γ-hemolysin), Exoenzymes (Proteases, Lipases, and Nucleases), and Toxins (Exfoliative toxins, Enterotoxins, TSST-1, and PVL

**Negative Regulation (−):** The *agr* system downregulates the expression of surface-associated proteins involved in host interaction, including Capsular polysaccharides, Protein A (Spa), Coagulase, Surface adhesion proteins (Fibronectin and Fibrinogen-binding proteins, Sbi, Clumping factor, Collagen, and Elastin-binding proteins).

### 3.2. The Staphylococcal Accessory Regulator (sar) System

The *Staphylococcal Accessory Regulator* (*sar*) system, particularly the SarA protein family, plays a crucial role in controlling the virulence of *S. aureus*, primarily by modulating genes regulated by the *agr* system. Foundational studies have demonstrated that SarA is essential for biofilm formation, toxin production, and immune evasion, contributing to the persistence of *S. aureus* in chronic infections [147,160]. The discovery of *sarV* as a transcriptional regulator repressed by SarA further illustrated the complexity of its regulatory network [161]. Additionally, SarA has been shown to enhance biofilm stability via the *Bap*-dependent pathway, reinforcing its importance in the bacterium’s ability to persist in harsh environments [162]. Mechanistic studies revealed that SarA autoregulates its expression, demonstrating the intricate feedback loops governing its regulatory functions [163,164]. The interplay between SarA and *agr* is further influenced by SarZ, which modulates both regulatory systems to fine-tune virulence expression [165].

Beyond its direct influence on virulence genes, SarA integrates metabolic signals into its regulatory framework, linking pathogenesis to bacterial metabolism [166]. Protein interaction studies have revealed how SarA collaborates with other regulatory proteins to control *agr* expression and other virulence determinants, adapting to environmental cues [167,168,169]. Identifying multiple SarA homologs within *S. aureus* suggests a more complex regulatory landscape influencing staphylococcal pathogenesis [135,170]. Moreover, recent findings have underscored the role of SarA in modulating toxin production during osteomyelitis, highlighting its contribution to bone infections through regulating extracellular proteases and virulence factors [78,171].

## 4. Antibiotic Resistance and Mechanisms in *S. aureus*

*S. aureus* causes various human infections, from mild cutaneous infections to severe diseases like pneumonia, endocarditis, and sepsis. One of its most concerning traits is its ability to develop resistance to multiple antibiotics, making treatment increasingly challenging [2,172]. The emergence of MRSA and vancomycin-resistant *S. aureus* (VRSA) is now one of the most significant threats to public health. *S. aureus* exhibits several mechanisms to resist antibiotic action, including enzymatic degradation of antibiotics, modification of target sites, efflux pumps, and biofilm formation [173,174].

### 4.1. Mechanisms of Antibiotic Resistance

*S. aureus* remains a formidable pathogen due to its remarkable ability to develop resistance to multiple antibiotics, posing significant challenges in clinical and community settings. The mechanisms underlying this resistance are diverse and multifaceted [175,176].

#### 4.1.1. Beta-Lactam Resistance

The emergence of resistance to β-lactam antibiotics in *S. aureus* is a crucial chapter in the history of AMR [176,177,178]. When penicillin was first introduced in the early 1940s, it was celebrated as a revolutionary medical breakthrough, demonstrating remarkable efficacy against bacterial infections, including those caused by *S. aureus* [179,180,181]. However, within a few years, reports of bacterial strains exhibiting resistance emerged. By 1942, isolated cases suggested that some *S. aureus* strains had become less susceptible to penicillin [175,182]. In 1944, more concrete laboratory evidence confirmed that certain strains could survive exposure to the antibiotic [183]. This growing concern culminated in a significant 1946 study by Kirby and colleagues, which documented the increasing prevalence of penicillin-resistant *S. aureus* in London hospitals. Their findings revealed that many bacterial isolates no longer responded to penicillin treatment, signaling a significant shift in the fight against bacterial infections [184,185,186].

As scientists investigated the cause of this resistance, they discovered that *S. aureus* had developed a mechanism to neutralize penicillin [186,187]. The key breakthrough came in the late 1940s when Chain and Abraham demonstrated that resistant strains produced an enzyme capable of breaking down the antibiotic [188]. This enzyme, initially called penicillinase and later categorized as a β-lactamase, was found to hydrolyze the β-lactam ring in penicillin, rendering it ineffective [181]. Further studies suggested that this resistance mechanism was genetically inherited, and researchers later identified the *blaZ* gene, often located on plasmids, as responsible for encoding β-lactamase [175].

The rapid emergence of penicillin resistance in *S. aureus* had profound implications for medicine. It demonstrated how quickly bacteria could evolve to evade antibiotic treatment, a phenomenon that continues to challenge healthcare today. The rise of resistant strains made previously treatable infections significantly more difficult to manage, leading to prolonged hospital stays and increased mortality rates [189]. Hospitals, where antibiotics were heavily used, became hotspots for resistant *S. aureus* strains, as the selective pressure created an environment that favored bacterial survival and resistance [176,190]. The discovery of β-lactamase provided a crucial understanding of how bacteria could render antibiotics ineffective, prompting researchers to develop new strategies to counteract resistance [191].

These early observations played a pivotal role in shaping the field of antimicrobial research. The realization that bacteria could rapidly adapt led to the development of new antibiotics designed to withstand enzymatic degradation [192,193,194]. Methicillin, a penicillinase-resistant antibiotic, was introduced in response to the rise of penicillin-resistant *S. aureus*. However, the subsequent emergence of MRSA demonstrated the persistent challenge of bacterial resistance and the ongoing need for innovation in antibiotic therapy [195,196].

#### 4.1.2. Mechanism of Methicillin Resistance in *S. aureus*

MRSA is predominantly mediated by the acquisition of the *mecA* or *mecC* genes, which encode alternative penicillin-binding proteins (PBPs) with reduced affinity for β-lactam antibiotics. The *mecA* gene, located on the staphylococcal chromosomal cassette *mec* (SCC*mec*), produces **PBP2a** (also termed PBP2′), a 668-amino-acid protein that replaces the transpeptidase function of native PBPs inhibited by β-lactams [9,195,197].

In MSSA, peptidoglycan synthesis relies on PBP1–4, which is inactivated by β-lactams through acylation of their transpeptidase domains. In MRSA, PBP2a maintains transpeptidase activity even under β-lactam exposure, enabling cross-linking of pentaglycan bridges between peptidoglycan chains [9,198].

The SCC*mec* element is a mobile genetic platform that integrates into the *orfX* locus of the *S. aureus* chromosome. It comprises the *mec* gene complex (*mecA/mecC*, regulatory genes *mecI* and *mecR1*), recombinase genes (*ccrAB* or *ccrC*), and joining regions (J1–J3) that may harbor additional resistance determinants [199]. *mecA* is hypothesized to originate from *Staphylococcus sciuri*, with horizontal transfer facilitating its incorporation into *S. aureus* [200]. A less common variant, *mecC* (70% homologous to *mecA*), encodes PBP2c, which exhibits higher oxacillin affinity than cefoxitin but reduced thermal stability at 37 °C, limiting its clinical prevalence [198].

Regulation of *mecA* expression involves the *mecR1-mecI* operon. In the absence of β-lactams, the repressor MecI binds the *mecA* promoter to suppress transcription. Upon antibiotic exposure, the sensor–transducer MecR1 undergoes autoproteolysis, cleaving MecI and derepressing *mecA*. Additionally, the anti-repressor MecR2 enhances *mecA* expression by disrupting MecI-DNA binding under stress [198,201]. Constitutive expression occurs in strains with Δ*mecR1-mecA* deletions [9,202].

Resistance extends to all β-lactams except ceftobiprole and ceftaroline, which retain activity against PBP2a [9,203]. SCC*mec* mobility, driven by Ccr recombinases, enables rapid dissemination of resistance across MRSA lineages, underscoring the role of horizontal gene transfer in antibiotic resistance evolution [199,204].

#### 4.1.3. Glycopeptide Resistance

The emergence of glycopeptide resistance in *S. aureus* has introduced significant challenges in clinical settings. Vancomycin, traditionally considered the ultimate treatment option for MRSA infections, has become less effective due to the evolution of resistance mechanisms [205,206]. Based on their minimum inhibitory concentration (MIC), *S. aureus* isolates are classified into three categories: vancomycin-sensitive (VSSA), vancomycin-intermediate (VISA), and vancomycin-resistant (VRSA) [207]. The first VRSA case was reported in 2002 in a patient with a catheter-related infection, where molecular analysis identified the presence of the *vanA* gene, a key determinant of vancomycin resistance [208,209].

The primary mechanism of vancomycin resistance in *S. aureus* involves modifications in peptidoglycan precursors that prevent vancomycin from binding effectively. Under normal conditions, vancomycin targets the D-Ala-D-Ala terminal of peptidoglycan precursors. However, resistant strains alter this site to D-Ala-D-Lac or D-Ala-D-Ser, drastically reducing vancomycin’s binding affinity [206,210,211]. These modifications are mediated by a cluster of resistance genes known as the *van* cluster, with *vanA* and *vanB* being the most frequently identified [175].

The *vanA* gene cluster, often acquired via horizontal gene transfer from vancomycin-resistant *Enterococcus* (VRE), is critical in conferring high-level resistance. Transmission occurs through conjugative plasmids, enabling the rapid dissemination of resistance among bacterial populations. The acquisition of these genetic elements presents a serious challenge in clinical settings, as VRSA strains exhibit high levels of resistance to vancomycin, rendering standard treatment regimens ineffective [208,212,213].

In contrast to VRSA, VISA strains do not acquire *van* genes but develop resistance through alternative mechanisms, primarily involving thickened cell walls and metabolic adaptations [8,214]. These strains exhibit excessive peptidoglycan synthesis, leading to increased cell wall density, which sequesters vancomycin molecules before they can reach their targets [215,216]. Additionally, VISA strains often harbor mutations in genes associated with cell wall biosynthesis and regulatory systems, such as *walKR* and *rpoB* [8,217,218]. The heterogeneity of VISA strains suggests that multiple genetic pathways play a role in resistance, making it challenging to develop targeted therapeutic approaches. Furthermore, VISA strains may serve as precursors to the eventual emergence of fully resistant VRSA strains, emphasizing the need for continuous surveillance and novel treatment strategies [216,219,220,221].

The increasing prevalence of VRSA and VISA strains underscores the urgency of developing alternative therapeutic strategies, including novel antimicrobial agents, combination therapies, and adjunctive treatments targeting resistance mechanisms. Enhanced infection control measures, rapid diagnostic tools, and antimicrobial stewardship programs are essential to mitigate the spread of vancomycin resistance in clinical settings [222,223].

#### 4.1.4. Aminoglycoside Resistance

The growing resistance of *S. aureus* to aminoglycosides presents a considerable challenge in medical environments, primarily due to the presence and function of aminoglycoside-modifying enzymes (AMEs). Research has progressively clarified the intricate mechanisms contributing to this resistance, mainly by identifying and analyzing AMEs and their genetic origins [224,225].

Specific AME genes, such as *aac(6′)-Ie/aph(2″)*, *ant(4′)-Ia*, and *aph(3′)-IIIa*, have been identified in MRSA isolates, playing a crucial role in conferring resistance to aminoglycoside antibiotics [226]. These genes encode enzymes that chemically modify aminoglycosides, rendering them ineffective. Their presence in MRSA strains complicates treatment options, particularly in hospital settings, where multidrug-resistant (MDR) infections pose significant therapeutic challenges [225]. Among these strains, SCC*mec* types II and V have been predominantly associated with hospital-acquired MRSA, suggesting a strong correlation between genetic background and resistance profiles. SCC*mec* type II is commonly linked to healthcare-associated infections and often carries additional resistance determinants, making MRSA strains more challenging to eradicate. SCC*mec* type V, while traditionally associated with CA-MRSA, has also been detected in hospital settings, indicating its potential role in the spread of resistance [227,228].

MRSA employs multiple mechanisms to evade aminoglycoside antibiotics. One strategy involves modifying the ribosomal binding site, preventing the drug from effectively inhibiting protein synthesis. Another mechanism reduces drug permeability, limiting intracellular antibiotic accumulation [173]. However, the most significant strategy is enzymatic inactivation, where AMEs chemically alter aminoglycosides, neutralizing their antimicrobial effects [175]. These resistance mechanisms highlight the intricate relationship between genetic determinants and the clinical challenges MRSA infections pose [229].

Aminoglycoside acetyltransferases (AACs), particularly AAC(6′), play a significant role in resistance by modifying aminoglycosides at the cellular level. Studies suggest that these enzymes may have evolved to interact with aminoglycosides due to their structural resemblance to natural cellular substrates. This discovery provides insight into the dual functions of AMEs, indicating that, beyond contributing to antibiotic resistance, they may also play a role in normal bacterial metabolism [230,231].

In addition to enzyme-mediated resistance, other resistance factors enhance MRSA’s ability to withstand aminoglycoside treatment. Reduced drug uptake, methylation of the 16S rRNA by methyltransferases, modifications to the 30S ribosomal subunit, and the activation of efflux pumps collectively contribute to bacterial survival in antibiotic-rich environments [232,233].

Given their significance in treating severe bacterial infections, the WHO classifies aminoglycosides as critically important antimicrobials. The emergence of AME-mediated resistance in zoonotic pathogens raises concerns about the spread of resistance across human and animal populations. Resistance genes are frequently shared among bacterial species, reinforcing the importance of continuous surveillance. Addressing these concerns requires a One Health approach, integrating human, animal, and environmental health strategies to monitor and mitigate the spread of resistance in diverse ecological settings [234,235,236,237].

#### 4.1.5. Fluoroquinolone Resistance

The increasing prevalence of fluoroquinolone resistance in *S. aureus*, particularly MRSA, presents significant challenges in clinical settings. The complexity of this resistance is driven by multiple factors, as demonstrated by nearly two decades of research into its underlying mechanisms [65,200,238].

Fluoroquinolones have become essential antibiotics for treating urinary tract infections and community-acquired pneumonia. However, their extensive use has contributed to the emergence of resistance. Underdosing and overuse have been identified as key factors accelerating this process. Identifying resistance mechanisms has been crucial in understanding how *S. aureus* adapts to fluoroquinolone exposure [239,240,241].

One major contributor to fluoroquinolone resistance is the emergence of mutations in the *grlA* and *gyrA* genes, which encode subunits of topoisomerase IV and DNA gyrase, the primary targets of fluoroquinolones [242]. Mutations in these genes reduce the drug binding affinity, diminishing the antibiotic’s effectiveness. Additionally, efflux pumps, such as NorA, play a crucial role by actively expelling fluoroquinolones from bacterial cells, further enhancing resistance. These efflux systems contribute to multidrug resistance in MRSA, complicating treatment strategies and reducing therapeutic options [243,244].

Global epidemiological studies have highlighted the widespread nature of fluoroquinolone resistance, particularly in hospital settings. Intensive care units are particularly affected, as MRSA strains exhibit high rates of resistance. The co-selection of resistance traits among different antibiotic classes further limits treatment options, emphasizing the need for revised clinical guidelines [245,246].

Genomic analyses have provided more profound insights into resistance evolution. Whole-genome sequencing has revealed specific mutations associated with fluoroquinolone resistance in pandemic MRSA clones, underscoring the role of selective pressure from antibiotic use in shaping resistance patterns. Notably, subinhibitory concentrations of fluoroquinolones have been found to accelerate resistance development, highlighting the importance of proper dosing in clinical practice [247,248].

Beyond genetic mutations and efflux pump activity, other resistance mechanisms contribute to fluoroquinolone tolerance in *S. aureus*. These include modifications to regulatory pathways that influence antibiotic susceptibility and metabolic adaptations that enhance bacterial survival in fluoroquinolones [249,250].

#### 4.1.6. Tetracycline and Macrolide Resistance

*S. aureus*’s resistance to tetracyclines and macrolide*s* is mediated by various genetic and biochemical mechanisms, enabling the bacterium to survive treatment with these commonly used antibiotics.

Tetracyclines, such as tetracycline and doxycycline, function by binding to the bacterial 30S ribosomal subunit, preventing the attachment of aminoacyl-tRNA and inhibiting protein synthesis [251,252,253]. Resistance to tetracyclines in *S. aureus* primarily occurs through active efflux pumps and ribosomal protection proteins. The most common efflux pump genes, *tet(K)* and *tet(L),* encode membrane-associated proteins that actively transport tetracycline out of the bacterial cell, reducing intracellular drug concentration and thereby limiting its efficacy [249,254,255]. Additionally, ribosomal protection proteins, such as those encoded by *tet(M)* and *tet(O)*, allow bacteria to continue translating proteins despite the presence of tetracyclines by preventing the drug from binding effectively to the ribosome [256,257,258]. These resistance determinants are frequently found on plasmids and transposons, facilitating their horizontal transfer among *S. aureus* strains, including MRSA isolates [65,254,259].

Macrolides, such as erythromycin, azithromycin, and clarithromycin, inhibit bacterial protein synthesis by binding to the 50S ribosomal subunit and preventing peptide elongation [260,261]. Resistance to macrolides in *S. aureus* is primarily mediated by target site modification, active efflux, and enzymatic inactivation. The most common resistance mechanism involves methylation of the 23S rRNA component of the 50S ribosome, encoded by the *erm(A)* and *erm(C)* genes, which confer resistance to macrolides, lincosamides, and streptogramin B (MLS_B resistance) by preventing macrolide binding [262,263,264,265,266]. Active efflux pumps, such as those encoded by *msr(A)*, expel macrolide molecules from the bacterial cytoplasm, reducing their intracellular concentration and efficacy [249,267]. Additionally, enzymatic inactivation of macrolides through esterases or phosphotransferases, although less common, has been reported in some *S. aureus* isolates [268,269].

### 4.2. Epidemiology and Clinical Impact of MRSA

#### 4.2.1. HA-MRSA

HA-MRSA is primarily found in hospitals, nursing homes, and long-term care facilities, where it predominantly affects patients with weakened immune systems, chronic illnesses, or those with invasive medical devices, such as catheters, ventilators, or surgical implants [7,270]. These strains are often classified as MDR due to their resistance to multiple antibiotic classes, including those beyond β-lactam antibiotics. These include aminoglycosides, fluoroquinolones, macrolides, and tetracyclines, which significantly limit therapeutic options and complicate treatment strategies [271]. The resistance mechanisms employed by HA-MRSA involve various genetic elements, including efflux pumps, target site modifications, and enzymatic degradation, making infections caused by these strains challenging to manage [9,272].

Clinically, HA-MRSA is a significant cause of severe healthcare-associated infections, such as bloodstream infections (bacteremia), ventilator-associated pneumonia, and surgical site infections. These infections often occur in immunocompromised patients, individuals with invasive medical devices (such as catheters or prosthetic implants), or those undergoing long-term hospitalization [273]. The severity of HA-MRSA infections is further compounded by their ability to evade host immune responses and persist in hospital environments, leading to recurrent outbreaks and increased morbidity and mortality rates [274]. The genetic basis of HA-MRSA resistance is attributed to the *SCCmec* types I, II, and III, which carry the *mecA* gene encoding penicillin-binding protein 2a (PBP2a). This protein alters cell wall synthesis, rendering β-lactam antibiotics ineffective [275].

#### 4.2.2. CA-MRSA

CA-MRSA, on the other hand, is more prevalent outside of healthcare settings and commonly infects otherwise healthy individuals. These infections are often seen in athletic teams, daycare centers, prisons, and military barracks, where close physical contact facilitates their spread. CA-MRSA strains differ from HA-MRSA in several important ways. CA-MRSA is generally less resistant overall, primarily exhibiting resistance to β-lactam antibiotics while remaining susceptible to many non-β-lactam agents such as clindamycin and trimethoprim-sulfamethoxazole [276]. A defining characteristic of CA-MRSA is its rapid spread in community settings, which is primarily attributed to the presence of the SCCmec type IV element. This genetic element is smaller and more mobile than in HA-MRSA, enhancing its ability to disseminate among individuals in environments like schools, sports teams, and correctional facilities [5]. Additionally, many CA-MRSA strains produce the PVL toxin, which contributes to severe necrotizing infections, including skin and *SSTIs* and necrotizing pneumonia [276].

#### 4.2.3. VRSA

VRSA has become an increasing concern in clinical settings due to its expanding prevalence. Since the first reported case in 2002, VRSA infections have been sporadically identified worldwide (Table 2), with most cases emerging in patients with chronic comorbidities, prior vancomycin exposure, and concurrent infections with VRE [208,277]. While the prevalence remains low, the potential for further adaptation and spread is a primary concern in healthcare settings.

Clinically, VRSA infections severely limit treatment options, as vancomycin has long been a cornerstone therapy for MRSA. Alternatives such as linezolid, daptomycin, and ceftaroline are available, but their efficacy varies depending on the infection site and resistance profile [278,279,280].

**Table 2:** Prevalence of VRSA in different countries.

Region	Prevalence (%)	Countries with Notable Data	Country-Specific Prevalence (%)	Reference
Asia	1.2%	India, Pakistan, Saudi Arabia	India (0.7%), Pakistan (0.1%), Saudi Arabia (18%)	[281]
Europe	1.1%	Italy, Turkey, Germany, France, Belgium	Italy (1.1%), Turkey (2.7%), Germany (0.7%), France (2.2%), Belgium (2.5%)	[281]
America	3.6%	Brazil	Brazil (3%)	[282]
Africa	2.5%	Ethiopia, Egypt	Nigeria (29%), Egypt (Multiple reports)	[222,283]
Middle East	-	Saudi Arabia, Egypt	Saudi Arabia (18%), Egypt (210 isolates reported)	[283]

## 5. Emerging Therapeutic Strategies for Mitigating *S. aureus* Biofilms, Antimicrobial Resistance, and Virulence Factors

### 5.1. Immunotherapeutic and Vaccination Strategies

Immunotherapeutic approaches combine precision targeting with adaptive immune activation to reduce antibiotic reliance against biofilm-associated infections. mAbs, such as TRL1068 and F598, neutralize adhesins (ClfA, FnBPs) and promote phagocytosis by resisting *S. aureus* proteases (e.g., GluV8) and evading Protein A (SpA) interference [284,285]. Multivalent vaccines (4C-Staph, Sta-V5) target surface antigens (IsdB, ClfA, CP5/CP8), inducing robust Th1/Th17 and γδ T-cell responses [284,286]. In contrast, passive immunization (AltaStaph, anti-PBP2a IgG) lowers bacterial loads in preclinical models [287]. Extracellular vesicle (EV)-based vaccines presenting Hla and MntC antigens stimulate IFN-γ-dependent immunity, and conjugate vaccines, such as SA4Ag, disrupt biofilm formation by blocking Agr QS [288]. Vaccines targeting wall teichoic acid (WTA) or deacetylated poly-N-acetyl glucosamine (dPNAG) reduce bacteremia by 54–91% in murine models [287].

### 5.2. CRISPR-Cas Systems for Genetic Targeting

CRISPR-Cas9 technologies disrupt biofilm-associated resistance and virulence genes. Phage-delivered systems target *mecA* and *blaZ*, resensitizing MRSA to β-lactams, while CRISPR interference (CRISPRi) silences *icaA* (essential for polysaccharide intercellular adhesin synthesis) without affecting bacterial viability [289,290,291]. Engineered phages synergize with lysostaphin, achieving ~4.7-log reductions in biofilm mass by targeting *icaA* and *tarO* [289]. Multiplex CRISPR systems eradicate MRSA in vivo by cleaving resistance plasmids and chromosomal loci, preserving microbiota integrity [292].

### 5.3. Phage Therapy, Endolysins, and Antimicrobial Peptides

Bacteriophages and engineered endolysins bypass biofilm-mediated resistance through enzymatic and lytic mechanisms. Phage StAP1 lyses 46.3% of clinical MRSA isolates [293], while endolysins (LysK, Exebacase) hydrolyze peptidoglycan, synergizing with antibiotics (e.g., vancomycin) to reduce MRSA populations by 90% [294,295]. Encapsulated phages in liposomes (e.g., Staphefekt SA.100) enhance stability, and phage-lysin-antibiotic combinations (e.g., Sb-1 + daptomycin) achieve <2 log10 CFU/mL reductions [296]. Antimicrobial peptides (AMPs), such as LL-37 and SAAP-148, disrupt membranes via lipid phase consolidation, showing efficacy in chronic wounds and periprosthetic infections [200,297].

### 5.4. Nanotechnology and Nanoparticle Applications

Nanoparticles enhance antibiotic delivery and biofilm destabilization. Silver (AgNPs) and zinc oxide (ZnO) nanoparticles generate Reactive Oxygen Species (ROS), disrupting matrix integrity, while pH-responsive nanocarriers improve drug penetration [200,298]. Ferumoxytol and cerium-coordinated gold nanoparticles (Ce-AuNPs) degrade extracellular DNA (eDNA) via hydroxyl radicals [297]. Photothermal therapies using gold nanoparticles or MoS_2_ nanosheets induce hyperthermia, destabilizing QS-regulated adhesins [297,299].

### 5.5. Biofilm Disruption: Enzymatic, Physical, and Metabolic Strategies

Enzymatic degradation of biofilms involves recombinant endolysins (XZ.700, ClyRODI-H5) and DNase I-functionalized nanogels, which degrade eDNA and Extracellular Polymeric Substances (EPSs) at ≤1 μg/mL [294,300]. Metabolic modulators like JBD1 increase NADH/ROS, sensitizing persisters to antibiotics, while Cu-POM nanoclusters disrupt the TCA cycle, inducing lethal metabolic stress [299].

Engineered endolysins (LysECD7-SMAP) and TiO_2_ photocatalytic coatings enhance biofilm penetration, enabling localized eradication [200,298]. Physical methods, including photothermal therapy and cryogenic freezing, destabilize matrix integrity, enhancing antibiotic penetration [297,301].

### 5.6. QS Inhibition

Natural compounds (berberine, thymol) and synthetic AIP analogs suppress the *agr* QS system, attenuating biofilm maturation [302]. Plant-derived flavonoids (punicalagin) and essential oils (cinnamaldehyde) disrupt EPS synthesis via oxidative stress, while probiotic-derived inhibitors enhance host–microbe competition [298,302,303].

### 5.7. Phytochemical and Small-Molecule Interventions

Phytochemicals (curcumin, resveratrol) inhibit NorA/QacA efflux pumps and staphyloxanthin biosynthesis, restoring antibiotic susceptibility. Small molecules (naftifine, NP16) repress α-toxin via ClpP protease targeting, achieving 90% biofilm eradication in combination therapies [304,305,306]

### 5.8. Anti-Virulence and Host–Pathogen Interaction Modulation

SpA-neutralizing mAbs counteract immune evasion, while bispecific antibodies (ClfA-Hla) synergize with antibiotics to prevent dissemination. Host-directed strategies enhance innate immunity via Th1/Th17 cytokines (IL-17, IFN-γ) and γδ T cells. *S. aureus* counters immunity through SSL3/TirS disruption of TLR2 signaling [307,308,309].

### 5.9. Combination Therapies and Surface Modifications

Dispersin B combined with vancomycin disrupts PIA, and lysostaphin-β-lactam combinations eradicate persister cells. Surface coatings (GL13K peptide, AGXX^®^) reduce bacterial adhesion by 40–94% on medical devices [298,300].

## 6. Conclusions and Future Directions

*S. aureus* remains a significant pathogen of global concern due to its remarkable adaptability, virulence, and ability to evade host immune defenses. The complex interplay between its extensive virulence factors, regulatory networks, and host interactions underscores the challenge of controlling *S. aureus*-associated infections. Advances in molecular microbiology, genomics, and artificial intelligence-driven approaches have significantly expanded our understanding of its pathogenic mechanisms, paving the way for novel diagnostic, therapeutic, and preventive strategies [272,310].

Despite these advances, several critical challenges remain. The rise of MDR *S. aureus* strains, particularly MRSA, seriously threatens public health. Understanding the evolutionary dynamics of antibiotic resistance and virulence factor expression is essential for developing more effective treatment regimens. Additionally, novel anti-virulence therapies, immunotherapies, and targeted antimicrobial strategies hold promise for mitigating *S. aureus*-related morbidity and mortality [200].

Future research should focus on deciphering the regulatory pathways governing virulence factor expression, identifying new therapeutic targets, and improving rapid diagnostic techniques. Integrating systems biology, omics technologies, and machine learning-based predictive models may provide deeper insights into *S. aureus* pathogenicity and AMR. Moreover, the development of vaccines remains a crucial goal, necessitating further investigation into host immune responses and antigenic targets [272].

In conclusion, tackling *S. aureus* infections requires a multifaceted approach combining fundamental research, innovative diagnostics, and novel therapeutic interventions. Strengthening global surveillance programs, promoting antimicrobial stewardship, and fostering interdisciplinary collaborations will mitigate the burden of S. aureus-associated diseases in future years.

## Figures and Tables

**Table 1:** Nasal carriage rates of MRSA and MSSA across different countries and populations.

Country	Population Studied	MSSA Carriage Rate	MRSA Carriage Rate	Source
United Kingdom	Orthopedic outpatients	22.4–35.6%	1.2–4.3%	[34]
USA	Patients undergoing fracture fixation	20.18%	4.70%	[35]
USA	Critically ill pediatric patients	Not specified	6.5%	[36]
Ethiopia	Hospital janitors	22.2%	8.1%	[32]
Ethiopia	Non-hospital janitors	14.4%	1.4%	[32]
Argentina	Healthcare workers	23.7%	6.3%	[37]
France	Healthy Blood Donors	29.3%	0.3%	[38]
France	Hospitalized Patients	20.2%	1.1%	[38]
Sierra Leone	General community	42.7%	14%	[39]
Lebanon	General community	38.4%	1.6%	[40]
China	Medical students	15.4–23.1%	3.0–9.4%	[41]
Saudi Arabia	Healthcare workers	40%	18%	[42]
Algeria	Livestock and humans in contact	50% (Humans)	7.6% (Livestock)	[43]
Portugal	Homeless individuals (Lisbon	50%	1.2%	[33]

## Data Availability

The data presented in this study are available within the article. Raw data supporting this study are available from the corresponding author upon reasonable request.
